# A ‘fit’ microbiota to potentiate cancer immunotherapy

**DOI:** 10.1186/s13073-015-0256-x

**Published:** 2015-12-21

**Authors:** Maria Rescigno

**Affiliations:** Dipartimento di Oncologia ed Emato-Oncologia, Universita’ degli studi di Milano, Immunotherapy Programme, European Institute of Oncology, Via Adamello, 16 20139 Milan, Italy

## Abstract

Cancer immunotherapy is very effective and leads to a long-term response in certain patients. Yet, the variability observed in this response indicates that additional factors related to the host must influence the activity of the treatments. Recent research suggests that the microbiota might play an important part in this variability.

## Linking the microbiota and immunotherapy

One observation that puzzles researchers is why the same tumor cell line injected into genetically identical mice gives rise to highly variable tumors. Why these tumors respond differently to therapy is also not clear. What is the difference between individual mice considering that their genome is identical? Two recent articles published in *Science* [1, 2] show that this difference can be attributed to the microbiota.

The microbiota is the community of microorganisms that inhabit all the surfaces in an organism that are exposed to the external environment, including the gut. The microbiota is involved in several host functions, including digestion of complex food macromolecules, behavior and the development of the immune system [3]. The microbiome (which is the genome of the microbiota) is 100 times larger than the human genome, and thus contributes a huge quantity of additional, acquired proteins and enzymes [4]. The microbiota is inherited from mothers during delivery and lactation and is subsequently shaped by diet and environmental factors [5, 6].

Immunotherapy is changing the treatment of patients with metastatic cancer and leads to a long-term response in a subset of patients [7]. Immune checkpoint inhibitors (ICIs), such as anti-CTLA4 and anti-PD-1/PD-L1 molecules, are being used effectively in clinical practice. These inhibitors reactivate T cells to ‘resurrect’ them from an ineffective state that does not allow them to respond to antigens. However, we do not know how T cells are reactivated and what the characteristics of the patients who respond are.

## Learning from mouse models

Vetizou et al. [1] and Sivan et al. [2] show that the efficacy of ICI treatment is dependent on the host microbiota. Indeed, in mice reared under germ-free conditions or treated with antibiotics the ICIs lost their therapeutic efficacy. In both studies, the authors found that, in the presence of the microbiota, host antigen-presenting cells activate interferon (IFN)γ-producing T cells, which are enriched during ICI treatment. It is amazing that the microbiota contributes to immune cell activation at distant sites and in particular tumor sites. The researchers excluded the possibility that these effects occur through systemic dissemination of the microbiota. This observation raises the question of whether microbial metabolites disseminate systemically and reach tumor compartments or whether these metabolites act on peripheral lymphoid organs.

In the study by Vetizou et al. [1], the researchers identified several *Bacteroides* species, including *Bacteroides fragilis*, and polysaccharide A produced by this bacterium as capable of promoting the maturation of intratumoral dendritic cells and inducing type 1 helper T cells in tumor-draining lymph nodes. Sivan et al. found that wild-type C57BL/6 mice from two different providers, the Jackson Laboratory (Jax) and Taconic (Tac), exhibited significant differences in the rate of melanoma growth, with tumors growing faster in Tac mice. The same trend was observed when mice were treated with anti-PD-L1, with a better response observed in Jax mice than in Tac mice. The authors compared the microbiotas of the mice housed in the two animal facilities and correlated their components with the amount of activated antigen-presenting cells in the tumor microenvironment. Only the levels of *Bifidobacterium breve*, *Bifidobacterium longum* and *Bifidobacterium adolescentis* were positively associated with the amount of antigen-presenting cells in tumors. Interestingly, administration of a mixture of *B. breve* and *B. longum* to Tac mice resulted in improved tumor control and increased IFNγ levels in tumor-draining lymph nodes and spleen.

## Microbial diversity and therapy outcome

These results indicate that having a ‘fit’ microbiota helps the immune system to perform effective immune surveillance. They also raise the questions of what a ‘fit’ microbiota is and how we can intervene to provide the best microbiota to patients. As the diversity of the microbiota is in part genetically determined [8], are some individuals predisposed to have a less effective microbiota, is the microbiota shaped during tumor development, or are both of these statements true?

Interestingly, Vetizou et al. [1] show that administration of *B. fragilis* or *Bacteroides thetaiotamicron* to wild-type mice can alter the activity of anti-CTLA4 in vivo, and also reduce the inflammatory response initiated by this antibody in the intestine. These findings indicate that the right bacterial combination can both potentiate the activity of ICIs and provide protection from the adverse effects of therapy, thus ‘uncoupling’ efficacy and toxicity of the antibody. The researchers also found that administration of anti-CTLA4 modifies the microbiota composition and increases the levels of the strains that seem to have a beneficial antitumor effect. These findings were paralleled by observations in patients with metastatic melanoma who were treated with anti-CTLA4. The researchers found that patients could be divided into three groups according to their microbiota (enterotypes) and that two enterotypes were associated with a better outcome than the other enterotype. The ‘good’ enterotypes were enriched in some, but not other, *Bacteroides* species that mediate the therapeutic effect of the drug, whereas the ‘bad’ enterotype still had quite a high number of *B. fragilis*, which potentiated the efficacy of anti-CTLA4 in mice. These findings suggest that either this species is effective only in the right microbial context or that some individuals select strains of *B. fragilis* that are more beneficial than others despite belonging to the same species.

It is obvious that these studies have huge therapeutic implications, but they also raise important issues. Can we improve an individual’s microbiota to achieve maximal therapeutic efficacy of immunotherapy? Is it sufficient to administer one species, such as *B. fragilis* or *B. breve*, or should we give a mixture of microorganisms, or even perform fecal transplantation of the microbiota? If two therapeutic options are available, should we select or exclude them according to the enterotype of the patient? As chemotherapy and cytokine-based immunotherapy also rely on the microbiota for their efficacy [9, 10], are there different enterotypes that mediate the response to different therapeutic agents or are there enterotypes that favor any possible therapy regardless of whether it is chemotherapy, immunotherapy or targeted therapy?

One thing is clear from these studies: the composition of our microbiota should be considered in future clinical studies aimed at assessing the therapeutic efficacy of new anticancer agents.

## Abbreviations

ICI: immune checkpoint inhibitor
IFN: interferon
